# Current Disease-Targets for Oleocanthal as Promising Natural Therapeutic Agent

**DOI:** 10.3390/ijms19102899

**Published:** 2018-09-24

**Authors:** Antonio Segura-Carretero, Jose Antonio Curiel

**Affiliations:** 1 Department of Analytical Chemistry, University of Granada, 18071 Granada, Spain; ansegura@ugr.es; 2 Functional Food Research and Development Center, Health Science Technological Park, 18016 Granada, Spain; 3 Torres Morente S.A.U., Bussines Park Metropolitano, 18130 Escúzar, Granada, Spain

**Keywords:** Oleocanthal, phenolic compounds, olive oil, therapeutic properties

## Abstract

The broad number of health benefits which can be obtained from the long-term consumption of olive oil are attributed mainly to its phenolic fraction. Many olive oil phenolics have been studied deeply since their discovery due to their bioactivity properties, such as Hydroxytyrosol. Similarly, in the last decade, the special attention of researchers has been addressed to Oleocanthal (OC). This olive oil phenolic compound has recently emerged as a potential therapeutic agent against a variety of diseases, including cancer, inflammation, and neurodegenerative and cardiovascular diseases. Recently, different underlying mechanisms of OC against these diseases have been explored. This review summarizes the current literature on OC to date, and focuses on its promising bioactivities against different disease-targets.

## 1. Introduction

The Mediterranean diet is characterized by a high consumption of olive oil, which plays a central role in the health benefits of the diet [1,2]. In fact, extra virgin olive oil (EVOO) in the Mediterranean region has long been associated with lower occurrences of certain chronic diseases, such as cancer incidence and cardiovascular mortality [3], as well as neurodegenerative dementias and Alzheimer disease [2,3,4,5,6]. The major components of olive oil are the fatty acids, of which the monounsaturated fatty acid (MUFA) oleic acid represents from 55% to 83% of the total fatty acids, polyunsaturated fatty acids (PUFA) from 4% to 20%, and saturated fat acids (SFA) from 8% to 14%. Other minor components of olive oil constitute from 1% to 2% of the total content, and are divided into two groups: i) the unsaponifiable fraction that could be extracted with solvents after the saponification of the oil, which contains squalene, triterpenes, sterols, tocopherol, and pigments, and ii) the soluble fraction that includes phenolic compounds [7].

Historically, the health benefits of virgin olive oil intake were attributed to the antioxidative properties of monounsaturated fatty acids (MUFAs), particularly oleic acid [5,6,8]. However, several seed oils (including sunflower, soybean, and rapeseed) containing high quantities of MUFAs are ineffective in beneficially altering chronic disease risk-factors [9,10].

A substantial number of investigations examined the biological functions of olive oil, suggesting phenolic compounds as being the beneficial constituents [2,11]. Those compounds found in EVOO have also been shown to bear antioxidant, anti-inflammatory, and anti-thrombotic activities; nevertheless, the exact mechanism of action remains unknown [5].

Although the cultivar, ripening stage, and geographic origin of olive and olive-tree irrigation can modulate the polyphenolic composition [12], the main phenolic compounds reported in EVOO are summarized in Table 1.

Tyrosol, hydroxytyrosol, and their secoiridoid derivatives represent around 90% of the total phenolic content of EVOO, which usually reaches concentration ranges between 100 and 300 mg/kg in EVOO [19,20], although concentrations as high as 500–1000 mg/kg have also been observed [21].

Dietary intake of olive oil polyphenols has been estimated to be around 9 mg, within 25–50 mL of olive oil per day, where at least 1 mg of them is derived from free tyrosol and hydroxytyrosol, and 8 mg are related with secoiridoid derivatives [22], in which oleocanthal (OC) is included, which is a popular and interesting phenolic compound whose beneficial bioactive functions are here reviewed.

## 2. Oleocanthal

**Figure 1:**
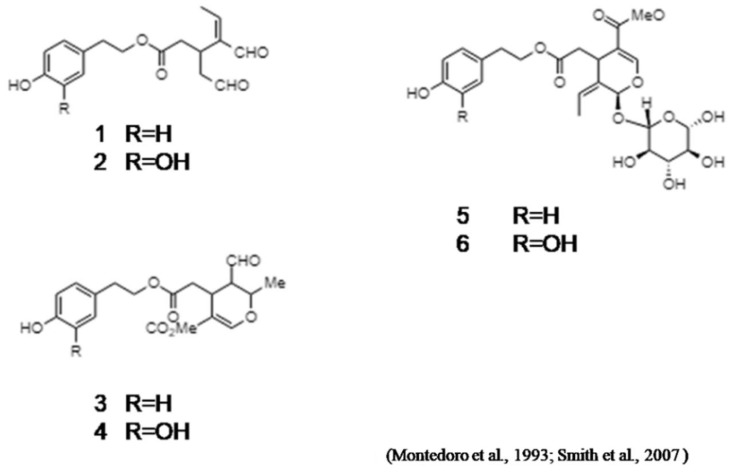
Oleocanthal (1) and related olive oil secoiridoids.

Montedoro et al. (1993) described the first isolation of secoiridoids from EVOO [19] (Figure 1). These secoiridoids comprise, in addition to OC, other important minor constituents which are implicated in the organoleptic properties of olive oils, including bitterness, pungency, and astringency [18].

Nevertheless, OC was subsequently identified by Busch and colleagues at Unilever Research and Development [18]. Concerning this fact, the structure of OC was assigned to both groups, employing a series of 1D and 2D NMR experiments [20,23] in conjunction with comparison to data in literature [19].

OC usually comprises about 0.02% by weight of EVOO [24], representing therefore about 10% of the total phenolic compounds [25,26]. However, OC concentration must be dependent on the olive variety and/or climatic conditions, since OC concentrations higher than 10% of the total phenolic compounds have also been described [27,28].

Chemically, OC is the elenolic acid ester of the common olive phenolic alcohol tyrosol [29] and is the principal molecule responsible for this pharyngeal pungency when EVOO is ingested [20,23].

That pungency was believed to signal potentially harmful compounds in our food, but consumption of many compounds eliciting this sensation, such as cinnamaldehyde and capsaicin, is also linked to decreased risks of cancer and degenerative and cardiovascular diseases [30,31]. However, unlike cinnamaldehyde, capsaicin, gingerol, and most known chemical irritants, OC does not significantly irritate the oral cavity; instead, the sting is restricted to the upper airways and is often accompanied by throat-clearing and coughing [2]. Those authors showed that OC activates the ion channel hTRPA1 ex vivo, and its ability to excite the trigeminal nervous system depends on functional TRPA1 in sensory neurons. In perceptual studies in humans, Peyrot des Gachonset al. (2011) observed that OC triggers irritation in the throat and nasal cavities with high potency compared with the anterior tongue, concluding that the high specificity of OC for the TRPA1 receptor and its restricted expression pattern characterizes the unusual pungency of extra-virgin olive oil [2].

## 3. Biological Effects of Oleocanthal

Despite hydroxytyrosol being described as the most potent phenolic antioxidant of olive oil and olive-mill waste water, which stimulated research on its potential role in cardiovascular protection [32], literature describes OC as the major phenolic compound in extra-virgin olive oil with broad functional and health benefitsthrough its capacity to interact with different specific disease targets, as is reviewed below.

### 3.1. Anti-Inflammatory Properties of Oleocanthal

OC’s anti-inflammatory properties was its first biological function described by Beauchamp et al. (2005) [23]. The hypothesis of those authors began with the similar pungencies found in both OC and solutions of the non-steroidal anti-inflammatory drug (NSAID), ibuprofen [33]. That idea made the authors think that pungency could be an indicator of similar pharmacological activities. Indeed, they observed that OC exhibited dose-dependent inhibition of the inflammatory cyclooxygenase enzymes COX-1 and COX-2 in vitro, and was more potent in inhibiting these inflammatory enzymes at equimolar concentrations in comparison to ibuprofen [23,34,35]. Beauchamp et al. (2005) also reported that 25 mM OC inhibited 41–57% of COX activity in comparison to 25 mM ibuprofen, which inhibited 13–18% COX activity in vitro [23].

**Figure 2:**
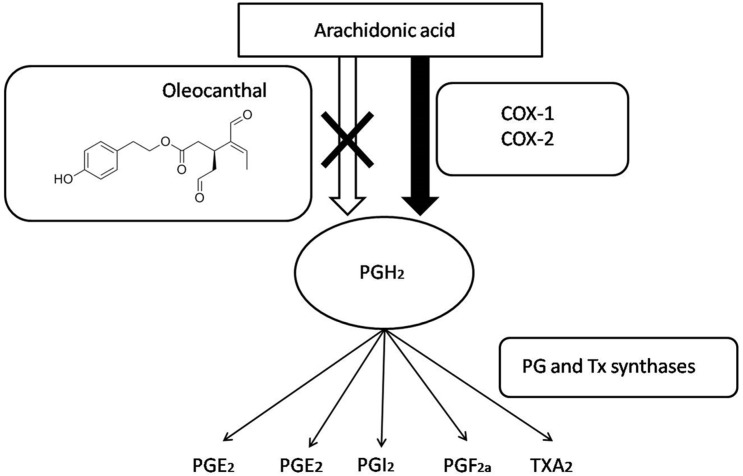
Prostaglandins, prostanoids, and thromboxane synthesis by the arachidonic acid pathway. OC and other NSAIDs inhibit both COX 1 and COX 2 enzymes blocking prostaglandin synthesis.

COX enzymes have their proinflammatory effectstriggered through the synthesis of prostaglandins and thromboxane (Figure 2), both starting from arachidonic acid [36,37]. That metabolism consists in the prostaglandin (PG) PGH_2_production via PGE synthase (PGES). PGH_2_ is a precursor of different prostanoids, including PGE_2_, PGI_2_, PGD_2_, PGF_2α_, and thromboxane (TXA_2_) [38]. Finally, prostanoids exert the inflammation process when they act on their receptors located on the surface of target cells to function [39].

Other inflammatory processes include the actuation of 5-lipoxygenase. This pro-inflammatory enzyme catalyzes the first steps in the biosynthesis of proinflammatory leukotrienes, and is therefore considered a promising drug target for the treatment of inflammatory diseases, such as asthma and allergic rhinitis [40]. These authors showed the importance of OC, as well as oleacein, for inhibiting 5-lipoxygenase through both antioxidant properties and the chelation of iron present in the active site of the enzyme [41]. Despite these properties being described for the most phenolic compounds [42], Vougogiannopoulou et al. (2014) demonstrated that both OC and oleaceinoffered better inhibition of 5-lipoxygenase [41].

Although OC constitutes approximately 10% of the total phenolic component of EVOO [25], this amount seems to be enough to contribute to the ability of olive oil phenolics to modify bodily physiological functions, potentially reducing risks for inflammatory disease [23].

### 3.2. Oleocanthal and Inflammatory Arthropathies

Osteoarthritis (OA) is the most common rheumatic disease and is a major cause of physical disability for elderly patients. It is characterized by progressive degradation that involves chondrocytes, cartilage, and other joint tissues, such as subchondral bone and the synovial membranes [33,43,44,45]. The OA aetiology is actually not completely understood [44]; nevertheless, ageing [46], female sex [46], obesity [47], or mechanical stress [48] are identified as OA risk factors.

The involvement of toll-like receptors (TLRs) in the innate immune response, as well as in the exacerbation of the inflammatory response and joint destruction in arthritis, has been postulated [49]. Concretely, the expression of TLR4 in cartilage increases throughout the development of OA [47].

Ligands for several of the TLRs have been identified, and usually correspond with microbial constituents, such as lipopolysaccharide (LPS), and also include nonbacterial products, such as Hsp-70 and fatty acids [49,50]. Ligand recognition by TLRs provokes a strong activation of pro-inflammatory cytokines, production of nitric oxide (NO), and up-regulation of costimulatory molecules which trigger the inflammatory process [51].

Despite nonspecific treatments for OA, such as NSAIDs and corticosteroids, they do not change the course of the disease and are even associated with adverse effects [45,47]. OC recently emerged as a potential therapeutic weapon for the treatment of inflammatory degenerative diseases because it blocks TLR4-dependent iNOS (inducible nitric oxide synthase) induction and TLR4 signaling by mouse chondrocytes [35].

On the other hand, nitric oxide (NO) is a highly reactive free radical and signaling molecule that plays a key role in inflammation, and is considered as a pro-inflammatory mediator that induces inflammation due to its over-production in abnormal situations [44,52]. Chondrocytes from patients with OA produce increased levels of NO, compared with those from healthy individuals [53]. Scotece et al. (2012) highlighted that OC suppresses lipopolysaccharide (LPS)-induced nitric oxide (NO) production in cultured J774 macrophages, and inhibits nitric oxide synthase gene expression [35]. Furthermore, these authors described that OC also inhibits expressionof the cytokines MIP-1α (macrophage inflammatory protein-1α) and IL-6 (interleukin-6), which are mediators of inflammation in rheumatic disease connected with OA in J774 murine macrophages and ATDC5 murine chondrocytes respectively, as well as the secretion of both cytokines in ATDC5 cells [35]. Because OC did not have any cytotoxic effect on J774 or ATDC5 chondrocyte cells [35,45], literature postulates that OC is a potent anti-inflammatory therapeutic agent for future treatment of arthritis or other inflammatory diseases.

### 3.3. Oleocanthal as Anti-Alzheimer Agent

Neurodegenerative diseases, including Alzheimer’s, are characterized by an increase of β-amyloid peptide oligomerization (Aβ), known as amyloid-derived diffusible ligands (ADDLs), which are a toxic species responsible for neurodegeneration [54], as well as abnormally hyperphoshorylatedtau proteins, causing neurofibrillary degeneration and therefore neuron cell death [55]. Several clinical studies highlight the use of non-steroidal anti-inflammatory drugs (NSAIDs) in the treatment of Alzheimer’s disease [56,57,58]. Indeed, ibuprofen and foods rich in polyphenols have been shown to attenuate the production of ADDLs and reduce tau proteins’hyperphosphorilation in animal models of Alzheimer’s disease [59,60,61,62,63].

**Figure 3:**
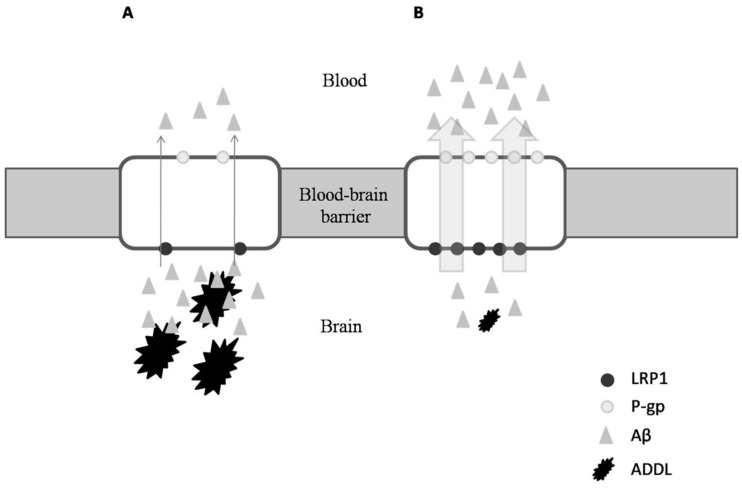
Transport of Aβ proteins through the blood–brain barrier under a situation of Alzheimer’s disease (AD) (**A**); after OC administration (**B**).

Due to the similar anti-inflammatory properties in ibuprofen and OC, Pitt et al. (2009) investigated the ability of OC also being an anti-Alzheimer agent [62]. These authors found that not only does OC disrupt Aβ oligomerization and therefore modify the state of ADDLs, it also has a neuro-protective effect in which OC appears to allow synapse-bound ADDLs to be more accessible to antibodies, thereby enhancing Alzheimer disease immunotherapy [62]. Similarly, Li et al. (2009) examined the neuroprotective effects that OC may possess [5]. Interestingly, these authors observed the in vitro inhibition of tau proteins’ fibrillization caused by OC when other NSAIDs, including ibuprofen, failed [5,62]. To this point, Abuznait et al. (2013) and Qosa et al. (2015) researched further into the beneficial effects of OC against Alzheimer’s disease, and reported its ability to induce the genes’ expression of P-glycoprotein (P-gp) and the LDL lipoprotein receptor-related protein-1 (LRP1) [63,64]. Both P-gp and LRP1 are the major Aβ transport proteins, and are responsible for amyloid clearance (Figure 3B). Similar results have been described in mouse models when OC was supplied with donezepil, a specific acetylcholinesterase inhibitor used for treatment of Alzheimer’s disease [65].

On the other hand, OC exhibited nonspecific covalent interaction with isomer 441 of the tau protein (tau-441), inducing a conformational rearrangement that could explain the antifibrillogenic ability of OC and could also account for a downregulation of the abnormal hyperphosphorylation of tau proteins [66]. A detailed analysis of the reactive profile of OC towards the tau protein—specifically, to fragment K18 of the tau protein—has been performed under biologically relevant conditions, giving new insights into the mechanism of interaction at the molecular level, such as the dependency of the temperature reaction and time of contact between the OC and tau [67].

### 3.4. Oleocanthal as Anticarcinogenic Agent

Several studies have reported different ways in which OC induces apoptosis and inhibits the migration, angiogenesis, and metastasis of cancerous cell lines originating from hepatocellular cancer [68], prostate cancer [68,69], human melanoma [70], non-melanoma skin cancer, [71], colorectal carcinoma [72], and breast cancer [73]. Although few in vivo studies have been reported on these thus far (Table 2), literature describes various interesting cancer targets for OC, being the main phenylethanoid studied for both the c-MET and hepatocyte growth factor (HGF).

The MET proto-oncogene encodes for the receptor tyrosine kinase, c-MET. Expression of c-MET is essential for embryonic development and tissue repair [76,77]. The hepatocyte growth factor (HGF) is the only known ligand for the c-MET receptor and is expressed mainly in cells of mesenchymal origin [78]. HGF and c-Met action is providential for tissue development by stimulating mitogenesis, morphogenesis, migration, and organization of 3D tubular structures, like renal tubular cells, cell growth, and angiogenesis [79,80]. However, several studies reported that deregulation or improper activation of the HGF/Met signaling pathway can promote cytoskeletal changes, leading to the acceleration of proliferation, angiogenesis, motility, and survival and invasive/metastatic abilities of many cancer cells [81].

Given the implications of c-Met for leading to the cancer-cell abilities previously listed, Elnagar et al. (2011) suggested the potential c-Met inhibitor of OC in silico due toits excellent binding affinity towards c-Met crystal structures [82]. In addition, antiproliferative, anti-migratory, and anti-invasive activities of OC were evaluated in vitro in different cancer cells such as MCF7 (nonmetastatic human breast cancer cells), MDA-MB-231 (highly metastatic human breast cancer cells), and PC-3 (human prostate cancer cells). That interesting study by Elnagar et al. (2011), of which results were subsequently confirmed in vivo by Akl et al. (2014), revealed the dose-dependent ability of OC to inhibit the proliferation, migration, angiogenesis, and invasion of the epithelial human breast and prostate cancer cell lines through the inhibition of c-Met phosphorylation [68,82].

Similar in vivo results of human hepatocellular carcinoma and human melanoma have recently been reported by Pei et al. (2016) and Gu et al. (2017), respectively [69,74]. Both scientific groups observed the inhibition of cancer cell migration by OC using a lung metastasis model. Interestingly, Pei et al. (2016) and Gu et al. (2017) described the potential of OC to block the activity, localization, and transcriptional activity of a novel cancer target, STAT3 [69,74]. In both hepatocellular carcinoma and human melanoma cancers, the STAT3 transcription factor leads to the survival, proliferation, invasion, and angiogenesis of human carcinoma by regulating the subsequent expression of target cancer genes involved [69,70,74]. Furthermore, in melanoma, STAT3 is constitutively activated and the high expression of phosphorylated STAT3 (p-STAT3) is associated with melanoma progression, and is required to enhance the invasive ability of cancer [82,83].

Gu et al. (2017) furthered the study about the effects of OC on STAT3 target genes, including myeloid leukemia cell differentiation (Mcl-1), vascular endothelial growth factor (VEGF), B-cell lymphoma-extra large (Bcl-xL), and matrix metalloproteinase-2 and -9 (MMP-2/9) genes [74]. They highlighted that OC inhibited the migration and invasion of A375 and A2058 human melanoma cell linesby downregulating the expression of MMP-2/9 [74] by the ability of OC to induce apoptosis in melanoma cells by inhibiting the expression of Bcl-xL and Mcl-1 [74] and reducing the expression of VEGF in melanoma cells, suggesting that the anti-angiogenesis effect of OC on melanoma is associated with VEGF inhibition [74].

Cusimano et al. (2017) reached similar conclusions as Pei et al. (2016) after OC treatment in different hepatocellular cell lines (HepG2, Huh7, Hep3B, and PLC/PRF/5) [84]. Moreover, they explored the in vitro OC ability to induce apoptosis of colorectal carcinoma in HT29 and SW480 cell lines [84]. However, those authors highlighted the dose-dependent capability of OC through inducing the expression of another cancer target, γH2AX, a marker of DNA damage, and increasing intracellular ROS production, causing mitochondrial depolarization of cancer cells [84].

Cutaneous Squamous Cell Carcinoma (cSCC) is an aggressive non-melanoma skin cancer, which originates from the spinous layer with a high probability of developing metastasis, and is responsible for most deaths associated with non-melanoma skin cancer [71]. The epidermal growth factor (EPG) is key when binding to its cognate receptor EGFR for leading to the activation of RAS/MEK/ERK and PI3K/Akt/mTOR pathways, and these play a key role in the molecular pathogenesis of cSCC [85,71]. In this regard, Polini et al. (2018), recently observed the in vitro effect of OC, among other phenolics, on the human epidermoid carcinoma cell line, A431 [71]. OC highly induced dose-dependent apoptosis on A431 cells after 72h of incubation through reducing the expression levels of B-Raf, phosphorylated-AKT (p-Akt), and phosphorylated ERK (p-Erk) targets, probably due to the changes induced by OC on Hsp-90 chaperone [85].

Furthermore, literature shows other different targets where OC can induce cancer cells death. In this sense, LeGendre et al. (2015) described novel anti-proliferative cancer cell properties of OC by inducing the lysosomal membrane permeabilization (LPM) target, which inhibits the acid sphingomyelinase and causes destabilization between proteins required for lysosomal membrane stability in cancer cells [72]. Permeabilization of lysosomes in cancerous cells causes the release of lysosomal hydrolytic enzymes into the cytosol, which leads to apoptosis (via mitochondrial outer membrane permeabilization and caspase activation) or necrosis (via cytosolic acidification) [86]. Moreover, it has been reported that luminal breast cancers (Luminal A, Luminal B, Triple negative/basal-like and HER2 type) are characterized by the expression of estrogen receptors [87], which are associated with a higher risk of local recurrence and metastasis [87,88]. In this regard, Ayoub et al. (2017) recently demonstrated that OC treatment suppressed growth of both luminal A and B breast cancer cell lines in a dose-dependent manner and retained its antiproliferative activity in luminal breast cancer cells in which cell growth was inhibited in media containing estradiol, as well as in mitogen-free media [72]. These novel features of OC were demonstrated through its ability to downregulatethe estrogen receptors in BT-474 breast cancer cells both in vitro and in vivo, and by suppressing the growth of hormone-dependent breast cancer [73].

In addition, OC also induced significant inhibition of the mammalian target of rapamycin (mTOR) of which abnormal activation supports the proliferation of breast cancer cells [89] among other cancers and neurologic diseases [90,91]. Despite mTOR having a crucial role in integrating signals from energy homeostasis, metabolism, stress response, and cell cycle [92], its abnormal activation is also involved in other pathogeneses, such as Alzheimer’s disease, where it increases the development of amyloid beta (Aβ) and tau proteins [90]. Furthermore, hyperactivation of the mTOR pathway by excessive food consumption is thought to be a critical factor which underlies diabetes [93]. Therefore, results described by Khanfar et al. (2015) reinforce the importance of OC as a therapeutic agent against Alzheimer’s disease and diabetes through its effects on the mTOR target [88].

On the other hand, Khanal et al. (2011) described the in vitro and in vivo anticarcinogenic effects of OC over adenosine monophosphate-activated protein kinase (AMPK) in HT-29 colon cancer cells [75]. AMPK is an interesting therapeutic target for cancer, well-known for its involvment in human cancer-cell apoptosis [94]. Although the literature shows that 5′-aminoimidazole-4-carboxamide-1-D-ribonucleoside (AICAR), metformin, and other phytochemicals, such as genistein, epigallocatechin gallate, and capsaicin, induce AMPK activation [95], Khanal et al. (2011) pointed out that in addition to activating AMPK, OC suppressed COX-2 expression and ledto the DNA fragmentation of HT-29 colon cancer cells, inducing their apoptosis [75]. Several studies have labeled the Hsp-90 chaperone as an important cancer target [96,97]. Hsp-90 is a key regulator of proteostasis under both physiological and stress conditions in eukaryotic cells. Hsp-90 chaperone is involved in many cellular processes beyond protein folding, which includes DNA repair, development, the immune response, and neurodegenerative disease, but it is also an essential chaperone crucial for the maturation of proteins such as Raf-1, ErbB2, actin, tubulin, and Cdk4, involved in cancer growth [97,98]. Also concerning this, Margarucci et al. (2013) suggested that OC can mediate both Hsp-90-ATPase activity inhibition and changes in the oligomerization of chaperones inducing cancer-cell inhibition [96]. Although those authors regrettably reported the inactivity of OC as an expression regulator of the Hsp-90 chaperone, Voiculescu et al. (2016), as well as Margarucci et al. (2013) proposed changes in the oligomerization of the Hsp-90 chaperone by OC, such as a mechanism to induce cancer-cell apoptosis [85]. Further studies are needed to clarify the role of OC on the Hsp-90 target.

### 3.5. Cardioprotective Effects of Oleocanthal

In the literature, there is currently only one study about the protective effects of OC against atherosclerotic cardiovascular disease (ACD). ACD is a chronic inflammatory disease initiated by endothelial damage and promoted by a number of cell types to include platelets [99].

In this regard, Agrawal et al. (2017) recently described that consumption of 40mL OC-rich EVOO (310mg of OC/kg oil) for one week increased the anti-platelet effects in healthy men aged between 20 and 50 years [100]. Although the extent of the response may be influenced by individual metabolism and/or diet, these beneficial effects best correlated with OC intake which reduced collagen-stimulated maximum platelet aggregation [100].

## 4. Future Prospects

Much evidence supports the protective effects of OC against a variety of major diseases. OC can offer interesting and different health benefits for a diverse series of illnesses. However, further in vivo studies in animal models and human trials should be designed to advance the research on OC’s health benefits, as well as to explore its beneficial effects on other diseases, such as obesity and metabolic syndrome, as have been described for other phenolic compounds.

Given that the concentration of OC usually comprises about 0.02% by weight of EVOO [24], some authors doubt the bioactivity of OC [25]. Although it is hard to determine the minimal dose for the biological effects of polyphenols in humans, further study on the concentration of OC, as well as its bioavailability, metabolism, and biological effects will be required.

Despite enrichment of OC in EVOO currently being applied, further efforts must be made to design novel, high-yield methods which allow OC to be obtained for future use in pharmacology. Some synthesis and extraction methods have already been described [6,101]; nevertheless, they are quite tedious, expensive, and have shown low OC yields.

In conclusion, the Mediterranean diet being regarded as a healthy one is directly relatedto its inclusion of EVOO. However, not all benefits can be assumed to be by OC, since different olive oil constituents have been characterized as being functional compounds, such as hydroxytyrosol, tyrosol, and oleuropein, among others. However, the pleiotropic bioactivities that OC promote go further than what the phenolics listed above can exhibit. Promising expected results will trigger the use of OC as a therapeutic agent in the near future.

## Figures and Tables

**Table 1:** Main phenolic groups and phenolic compounds identified in extra virgin olive oil (EVOO).

Polyphenolic Groups	Characteristics	Phenolic Compounds	References
Phenolic acids	Based on the chemical structure of C6–C1 for benzoic acids and C6–C3 for cinnamic acids derivatives	gallic acid, vanillic acid, caffeic acid, syringic acid, o-coumaric acid, protocatechuic acid, *p*-hydroxybenzoic acid, sinapic acid	[13]
Phenolic alcohols	Showing a hydroxyl group attached to an aromatic hydrocarbon group	hydroxytyrosol, tyrosol	[14]
Secoiridoids	Characterized by the presence of either elenolic acid or elenolic acid derivatives	Oleuropeinaglycone, demethyloleuropein, ligstrosideaglycone, nuzenide	[13,15]
Hydroxy-isocromans	Constituted by 3,4-dihydro-1H-benzo[*c*]pyran derivatives	1-(3-methoxy-4-hydroxy)phenyl-6,7-dihydroxyisochroman, 1,phenyl-6,7-dihydroxy-isochroman	[13,16,17]
Flavonoids	Characterized by two benzene rings joined by a linear three carbon chain. Sometimes glycosilated. They can be further divided into flavones and flavanols	apigenine, luteoline, (+)-taxifoline, rutin, luteolin-7-glucoside, glycosides of delphinidin	[16]
Lignans	The structure is based on aromatic aldehydes condensation	pinoresinol (P), 1-acetoxypinoresinol, hydroxypinoresinol	[18]

**Table 2:** Characteristics of selected in vivo studies showing anti-cancer effects of oleocanthal.

Animal Model	Damaging Agent	Treatment	Duration	Oleocanthal Cancer Target	Effects	Reference
Nude mice	Injection of 5 × 10^6^ A375 cells in 200 μl of PBS. Human melanoma	Oleocanthal or DMSO 15 mg/kg/day	1 week	Signal transducerand activator of transcription 3 (STAT3)	Significant decrease of tumor size. Ki-67 and CD31, markers of proliferation and angiogenesis respectively, were significantly decreased	[74]
Athymic nude mice	Injection of 1 × 10^6^ MDA-MB-231/GFP cells Human breast cancer	Oleocanthal or DMSO 5 mg/kg/day	4 weeks	HGF and c-Met	Reduction of 60% in tumor growth. Ki-67 and CD31 markers were significantly decreased	[68]
Male BALB/c athymic nude mice	Injection of 4 × 10^6^ HCCLM3-luc cells in 150 μL of PBS Human hepatocellular Carcinoma	Oleocanthal or DMSO 5 or 10 mg/kg/day	5 weeks	Signal transducerand activator of transcription 3 (STAT3)	Tumor gross reduction Ki-67 marker was decreased Increasing of apoptotic cells in a dose dependent manner	[69]
Fertilized chicken eggs	Injection of 2 × 10^6^ HT-29 cells Human colon carcinoma	Oleocanthal or saline solution (50 µg/mL)	3 days	Cyclooxygenase-2 (COX-2) and Adenosine Monophosphate-activated Protein Kinase(AMPK)	HT-29 cells inhibition AMPK significantly induced	[75]
Female athymic nude mice	Injection of 1 × 10^7^ BT-474 cells Human luminal breast cancer	Oleocanthal or DMSO 5 or 10 mg/kg/day	>8 weeks	Estrogen receptors α (ERα)	Significant reduction in tumor growth and volume	[73]

## Abbreviations

Aβ	β-amyloid peptides oligomerization
ADDLs	Amyloid-derived diffusible ligands
EVOO	Extra virgin olive oil
HGF	Hepatocyte growth factor
LMP	Lysosomal membrane permeabilization
mTOR	Mammalian target of rapamycin
MUFA	Monounsaturated fatty acid
NO	Nitric oxide
NSAID	Non-steroidal anti-inflammatory drug
OA	Osteoarthritis
OC	Oleocanthal
PG	Prostaglandin
PUFA	Polyunsaturated fatty acids
SFA	Saturated fat acids
TLRs	Toll-like receptors
